# The Influence of Parental Mindfulness on Preschool Child Behavior Problems: A Chain-Mediated Model Analysis

**DOI:** 10.3390/ijerph20010812

**Published:** 2023-01-01

**Authors:** Hehong Quan, Jingyu He, Chun Li

**Affiliations:** 1 Graduate School of Humanities and Social Sciences, Hiroshima University, Hiroshima 7398511, Japan; 2 Department of Preschool Education, Qingdao University, Qingdao 266000, China

**Keywords:** parental mindfulness, preschool child behavior problems, marital satisfaction, coparenting

## Abstract

This study aimed to examine the associations between parental mindfulness and preschool child behavior problems and to investigate the mediating role of marital satisfaction and coparenting in the relationship between mindfulness and preschool child behavior problems. This was a cross-sectional study in which general sociodemographic data were obtained from 3448 parents of children in grades junior to senior of kindergarten who were assessed using instruments such as the Mindful Attention Awareness Scale (MAAS), the Strengths and Difficulties Questionnaire (SDQ), the ENRICH Marital Satisfaction Scale (TEMSS), and the Parents’ Perceptions of the Coparenting Relationship Questionnaire (PPCR). (1) Mindfulness, preschool child behavior problems, marital satisfaction, and coparenting were significantly correlated with each other, where mindfulness was positively correlated with marital satisfaction and coparenting and significantly negatively correlated with preschool child behavior problems. (2) Mindfulness negatively predicted preschool children’s problem behaviors. (3) Marital satisfaction independently mediated the relationship between mindfulness and preschool child behavior problems. (4) Coparenting also independently mediated the relationship between mindfulness and preschool child behavior problems. (5) Marital satisfaction and coparenting play a chain-mediating role between mindfulness and preschool children’s problem behaviors. Mindfulness predicted preschool child behavior problems, with marital satisfaction and coparenting as mediators.

## 1. Introduction

The preschool stage is a period of rapid emotional and social development for young children, and because their physical and psychological functioning is still immature, they are prone to deviant behaviors [1] and even problem behaviors [2]. Achenbach and Edelbrock (1991) [3] divided problem behaviors into internalizing problem behaviors (e.g., anxiety, withdrawal) and externalizing behavior problems (e.g., aggression, lying). Although some behavior problems may gradually improve as children mature mentally, some researchers have noted that the development of problem behaviors in early childhood showed stability to an extent [4] and predicted lower academic achievement, more peer problems, and social maladjustment in adolescence during school age [5,6], requiring timely attention and guidance from adults.

For preschool children, the family setting is a key environmental factor affecting their development [7]. Family systems theory suggests that the behavior of each family member is the result of the joint influence of other family members [8]. Exploring the mechanisms that explain the formation of preschool children’s problem behaviors from the perspective of the interaction between family members has become crucial for the effective prevention and reduction of problem behaviors in preschool children.

In recent years, researchers have begun to focus on the effects of parental mindfulness on child development. Mindfulness refers to an individual’s purposeful, nonjudgmental attention to internal and external stimuli in the present moment [9] and is expressed as a psychological state or individual trait. Individuals with higher levels of mindfulness tend to be more adept at regulating their negative emotions and able to adopt positive coping strategies in the face of difficulties and setbacks [10,11]. Thus, higher levels of mindfulness in mothers tend to predict fewer behavior problems and positive social adjustment in children [12,13]. This is because mindfulness enhances mothers’ receptive attention and reduces impulsive responses [14], protecting parent–child intimacy and, hence, reducing the likelihood that preschool children will develop problem behaviors as a result of unmet psychological needs. Furthermore, parental mindfulness may also influence children’s levels of mindfulness through the effect of intergenerational transmission [15]. Research has found that young children with higher levels of mindfulness have stronger control abilities [16] and are less likely to develop behavior problems. Hence, this study proposes the following first hypothesis: parental mindfulness inversely predicts problem behaviors in preschool children.

Existing research on mindfulness also suggests that individuals with high levels of mindfulness tend to perform better in establishing and maintaining interpersonal relationships and are more likely to have satisfying marital relationships [17,18]. On the one hand, high levels of mindfulness are associated with a wide range of positive psychological outcomes, and these positive outcomes may influence an individual’s satisfaction with interpersonal relationships and, in turn, the functioning of romantic relationships. In a sample of 104 married adults, Jones, Welton, Oliver, and Thoburn (2011) [19] found that mindfulness was positively associated with relationship satisfaction. This may be related to the sharper insight [17], more active acceptance [20], stronger emotional communication ability [21], and empathy [22] associated with high mindfulness. On the other hand, mindfulness can also attenuate the negative effects of insecure attachment on relationships [23]. This is important for maintaining marital satisfaction, as insecure attachment is also a key risk factor for relationship breakdown [24] and reduced marital satisfaction.

Family systems theory suggests that subsystems in families are interdependent and that behaviors and emotions experienced by parents in the couple subsystem spill over [25,26] into the parenting subsystem, directly or indirectly influencing children’s problem behaviors. When parents are dissatisfied with their marital relationship, they are more likely to develop negative emotions and behaviors that spill over into their interactions with their children [27]. Studies have confirmed that children exposed to marital conflict develop more internalizing and externalizing problems, such as depression, anxiety, and aggression [28,29]. Hence, this study proposes hypothesis two: marital satisfaction is a mediating variable for the influence of parental mindfulness on preschool children’s problem behaviors.

Parenting preschool children is more challenging for parents than during their infancy [30], and thus the quality of coparenting between parents may be another important factor influencing children’s behavior problems [31]. Unlike marital satisfaction in the couple subsystem, coparenting spans the parenting subsystem [32] and reflects the extent to which parents support each other’s parenting behaviors in raising their children [33]. Negative coparenting between parents tends to cause anxiety and depression in preschool children [34], and further findings suggest that conflictual and demeaning coparenting has not only immediate effects on problem behaviors in adolescent children but also long-term, persistent effects [35]. The present study concluded that mindfulness concentrating on “awareness and acceptance” [9] is expected to enhance the quality of coparenting between parents because mindfulness focuses not only on the intrapersonal process but also the interpersonal process. In coparenting, individuals may show more accepting attitudes toward themselves and their partners [36]; stronger self-regulation, emotional regulation, and empathy may also help them participate more actively in coparenting [17]. Hence, this study proposes hypothesis three: coparenting is an additional mediating variable for the influence of positive parenting on preschool children’s problem behaviors.

Family factors that influence children’s development do not operate in isolation. There is an interactive joint effect between them [8], namely, parental mindfulness may also jointly influence preschool children’s problem behaviors by achieving marital satisfaction and coparenting. Studies have shown that strong mindfulness is associated with increased relationship satisfaction [37,38] and that there is a spillover effect between marital satisfaction and coparenting [39]. Hence, this study proposed Hypothesis 4: Marital satisfaction and coparenting play a chain mediating role in the effect of parental mindfulness on preschool children’s problem behaviors.

In summary, the acute awareness and acceptance that mindfulness brings may improve the parenting process and predict behavior problems in preschool children in reverse. This process may be achieved through mindfulness, increasing marital satisfaction, and improving coparenting. This study constructs a chain mediation model based on family systems theory to examine the mechanisms of marital satisfaction and coparenting in the relationship between parental mindfulness and preschool children’s problem behaviors. These findings will facilitate our understanding of *how* parental mindfulness influences preschool children’s problem behaviors and *what* we can do to prevent and mitigate such behaviors.

## 2. Methods

### 2.1. Participants

In this study, a convenience sampling method was used to distribute questionnaires to parents of junior, middle, and senior classes in 12 kindergartens in Shandong, Inner Mongolia, and Jilin, China. A total of 4000 questionnaires were distributed, and after excluding 552 of them owing to incomplete responses or responses with regular patterns, 3448 (86.2%) questionnaires remained valid. Of these, 1761 (51.07%) were parents of boys, and 1687 (48.93%) were parents of girls. The number of parents of junior-class students was 1193 (34.6%, children aged 3.5 to 4.5), middle-class students was 1010 (29.29%, children aged 4.5 to 5.5), and senior-class students was 1245 (36.11%, children aged 5.5 to 6.5).

### 2.2. Measures 

#### 2.2.1. Demographic Information

The questionnaire included a demographic information sheet that collected information, including whether the participant’s child was an only child, the participant’s occupation, education level, and average monthly household income. Details are shown in Table 1.

#### 2.2.2. Mindful Attention Awareness Scale (MAAS)

In this study, the MAAS, developed by Mackillop and Anderson (2007) [40] and revised by Chen et al. (2012) [41], was applied to investigate the level of mindfulness in parents. The scale contains fifteen items and is scored on a six-point Likert scale. Parents were asked to choose the level of each item that best matched their own description of actual occurrences in the most recent month (including that day), ranging from one “almost always” to six “almost never” according to the frequency. The higher the total score, the stronger the parent’s mindfulness in daily life. Cronbach’s alpha coefficient for the scale was 0.93 for this measurement.

#### 2.2.3. Strengths and Difficulties Questionnaire (SDQ)

In this study, the SDQ for preschool children, developed by Goodman (2001) [42] and revised by Du et al. (2008) [43], was used to evaluate preschool children’s problem behaviors. The scale, which was completed by parents who had knowledge of their child’s situation based on the last six months of the child’s life, consists of 28 items (five of which are reverse-scored: 7, 11, 14, 21, and 25) and includes five subscales, four of which were used in this study to evaluate the children’s problem behaviors (emotional, conduct, hyperactivity, and peer interaction problems). The scales were measured using a three-point Likert scale, with one representing “not true” and three “certainly true.” The higher the total score on the four subscales, the higher the level of problem behavior. The Cronbach’s alpha coefficient for the problem behavior subscale measurement was 0.76.

#### 2.2.4. The ENRICH Marital Satisfaction Scale (TEMSS)

The study used TEMSS, developed by Fowers and Olson (1993) [44] and revised by Li et al. (1993) [45], to assess parents perceived marital satisfaction. There are ten items on this scale, measured using a five-point Likert scale, where one represents “strongly disagree” and five “strongly agree.” This questionnaire can be used to measure the binary dimension [44]. For example, “we” in “I am very happy about how we make decisions and resolve conflicts” refers to “you and your spouse,” and can be a questionnaire indicator to measure the binary dimension. Certain questions (2, 4, 6, 7, and 10) were reverse-scored, and a mean score was calculated for all the scale items. The higher the score of marital satisfaction of one of the parents, the better the marriage’s quality. The Cronbach’s alpha coefficient for this measurement was 0.90. 

#### 2.2.5. Parents’ Perceptions of the Coparenting Relationship (PPCR)

This study used the PPCR questionnaire developed by Stright and Bales (2003) [46] and revised by Hou (2007) [47] to measure the extent to which parents perceive spousal support in child-rearing behaviors. The fourteen-item scale is divided into two dimensions: supportive coparenting (e.g., “My spouse supports me when I discipline my child”) and unsupportive coparenting (e.g., “My spouse does not help me when I need their help with child-related matters”), each with seven items, scored on a five-point Likert scale, with one being “never” and five “always.” The “unsupportive coparenting” dimension was reverse-scored, and the responses were combined with those of the “supportive coparenting” dimension to calculate the total mean score. The Cronbach’s alpha coefficient was 0.92 for this measurement.

### 2.3. Data Analysis

The data analysis for this study was performed using SPSS 25.0. Following the recommendations of Podsakoff et al. (2003) [48], we performed a common method bias test before conducting descriptive statistical and correlation analyses. Finally, we performed a chained mediating effects test using Hayes’s PROCESS macro (Model 6), with all analyses controlled for covariates.

## 3. Results

### 3.1. Common Method Bias Testing

In this study, data were collected uniformly in the form of a self-statement questionnaire for each research variable; thus, there may be a degree of common method bias that requires testing before data analysis to determine whether common method factors could have a significant effect on the results. As suggested by Zhou and Long (2004) [49], first, we controlled the data collection process by using methods such as anonymous completion and the reverse presentation of certain question items. To further improve the rigor of the study, the Harman single-factor test was used to test for common method bias [48]. The statistical results analyzed a total of nine common factors with a cumulative variance accounting for 53.18%, while the percentage of variance explained by the first common factor was 22.93%, well below the 40% criterion, indicating that there was no serious common method bias in the data.

### 3.2. Preliminary Analysis

The mean, standard deviation, and correlation matrix for each of the study variables are shown in Table 2. The results revealed significant correlations between parental mindfulness, marital satisfaction, coparenting, and preschool child problem behavior. In particular, parental mindfulness was significantly negatively correlated with problem behavior scores and its subdimensions while being significantly positively correlated with marital satisfaction and coparenting scores. Marital satisfaction was significantly positively correlated with coparenting and significantly negatively correlated with preschool child problem behavior and its subdimensions. The coparenting scores were significantly negatively correlated with preschool child problem behavior and its subdimensions.

### 3.3. Multiple Mediating Model Analysis 

**Figure 1:**
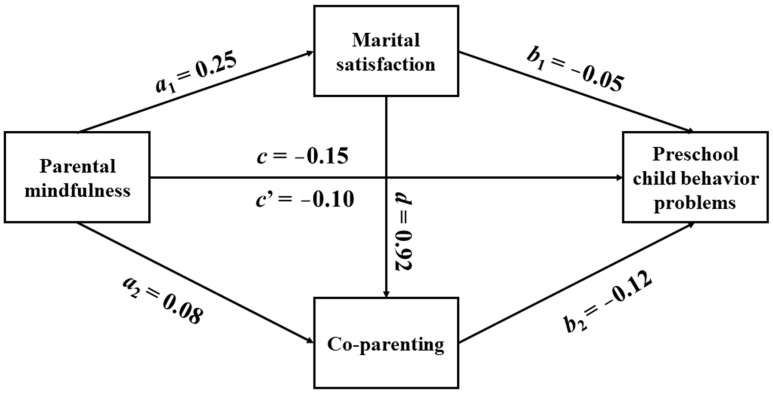
The pathway of the mediating model.

Based on these results, we tested the mediation model using SPSS macro PROCESS (Model 6) with 5000 bootstrap samples. The results are shown in Table 3 and Figure 1. The regression analysis showed that after controlling for demographic variables such as gender, grade level, parental occupation, parental education, and household economic income, parental mindfulness significantly negatively predicted preschool child problem behavior (*c*), while it was a significant positive predictor of marital satisfaction (*a*_1_) and the level of coparenting (*a*_2_). Marital satisfaction significantly positively predicted the level of coparenting (*d*) and negatively predicted preschool child problem behavior (*b*_1_). Parental mindfulness (*c*’) and the level of coparenting (*b*_2_) were significant negative predictors of preschool child problem behavior.

The mediation analysis (as shown in Table 4) revealed a significant mediating effect of marital satisfaction and coparenting in the relationship between mindfulness and preschool child problem behaviors (total indirect effect = −0.049, SE = 0.003, bootstrap 95% CI: [−0.056, −0.042]), accounting for a total effect of (−0.146) and a ratio of 33.56%. Meanwhile, the direct effect pathway (*c*’) between parental mindfulness and preschool child problem behavior was significant (direct effect = −0.097, SE = 0.006, bootstrap 95% CI: [−0.110, −0.084]), suggesting that marital satisfaction and coparenting play a partially mediating role in the effect of mindfulness on preschool child problem behavior. The mediating effect consisted of three mediating pathways: an independent mediating effect for marital satisfaction (*a*_1_*b_1_, mediating effect = −0.011, SE = 0.004, bootstrap 95% CI: [−0.019, −0.004], 7.53% of total effect), an independent mediating effect for coparenting (*a*_2_*b_2_, mediating effect = −0.010, SE = 0.002, bootstrap 95% CI: [−0.013, −0.007], 6.85% of total effect), and the chain mediating effect of marital satisfaction and coparenting (*a*_1_*d*b_2_, mediating effect = −0.028, SE = 0.003, bootstrap 95% CI: [−0.034, −0.022], 19.18% of total effect).

## 4. Discussion

### 4.1. The Effect of Parental Mindfulness on Preschool Child Problem Behavior

Results showed that parental mindfulness was negatively correlated with preschool child problem behavior, and the results not only supported Hypothesis 1, but were also consistent with the findings of previous studies [12,50]. Maternal mindfulness has been found to indirectly influence children’s internalizing and externalizing behavior problems through mindful parenting as well as positive and negative parenting behaviors in a sample of children aged 6–12 years [51]. The findings of the present study on a group of preschool children extend the age range of existing research, thereby providing new empirical evidence for improving preschool child behavior problems.

Duncan et al. (2009) [52] suggested that parents with strong mindfulness tend to be more sensitive to their own and their children’s emotions, more willing to actively listen and respond to their children’s needs, and more able to regulate themselves and their parent–child interactions in parenting such that their children acquire positive interpersonal skills and develop fewer behavior problems. Moreover, mindfulness can reduce negative outcomes for parents and children in parenting through six mechanisms: (1) reducing parental stress; (2) reducing parents’ attention to negative thoughts; (3) improving executive functioning in parents, especially for impulsive parents; (4) disrupting the intergenerational transmission of bad parenting habits; (5) increasing self-nourishing attention; and (6) improving marital relationships and functioning [53].

Another interesting perspective is that although parenting children with problem behaviors is challenging for most parents, it does not affect different parents to the same degree [54]. Research has shown that if parents have lower levels of mindfulness, they may tend to perceive their child’s problem behaviors as difficult to deal with, and are therefore more likely to report more severe problem behaviors [55], whereas parents with higher levels of mindfulness may tend to perceive their child as having fewer behavior problems because they have a higher mental capacity to cope [56]. It is evident that both parental mindfulness, which affects parents’ perceptions of their children’s problem behaviors, and parent–child relationship patterns have important implications for preschool child problem behavior.

### 4.2. The Chain Mediating Role of Marital Satisfaction and Coparenting in the Relationship between Parental Mindfulness and Preschool Child Problem Behavior

This study also found that marital satisfaction and coparenting mediated independently between parental mindfulness and preschool child problem behavior, respectively. In other words, hypotheses two and three are valid.

Gottman and Notarius (2002) [57] found that the marital satisfaction of couples declined each year after the birth of the first child. Children, as new members of the family, are constantly “setting” increasing challenges for their parents, and because of this, parents of preschool children are less satisfied with their marriage than those of infants [58]. Fortunately, if parents have a high level of mindfulness, they may be more likely to have a satisfying marital relationship because the awareness and acceptance that come with mindfulness increase positive emotions and enhance happiness [17,59]. Without the need for verbal transmission, a harmonious marital relationship as an important spiritual environment will influence children’s psychological need for safety and love, thereby preventing and reducing the development of problem behaviors.

It is undeniable that parenting is a joint activity for couples and that the quality of coparenting also influence children’s problematic behavior. At the core of mindfulness is non-judgmental acceptance, an attitude that is very important to coparenting. When one partner is able to receive acceptance, acknowledgement, and well-meaning advice from their partner, they develop more feelings of being supported; supportive coparenting influences the pattern of the parent–child relationship, reducing negative influences and enhancing the quality of parenting [4]. Conversely, if one partner perceives that their spouse is not supportive of their parenting, they will spend more energy dealing with negative emotions, and the negative state will spill over into the parent–child system and have an impact on the child’s problem behaviors.

Due to the spillover effect [27], marital satisfaction in the couple subsystem spills over to the parent subsystem and affects the quality of coparenting. Our study found that marital satisfaction and coparenting play a chain mediating role between parental mindfulness and preschool child problem behavior. Thus, hypothesis four is valid. Higher levels of parental mindfulness are associated with greater marital satisfaction in couples, which contributes to higher-quality coparenting (more supportive coparenting), thereby preventing and reducing the development of problem behaviors in preschool children. The results of chain mediation are consistent with the main ideas of family systems theory, which suggests that individual development is nested in a series of interacting family relationships [8]. From this perspective, the interrelationships between family members (marital satisfaction and coparenting) will have a direct or indirect effect on children’s problem behaviors.

## 5. Limitations and Directions for Future Research

First, this study did not distinguish whether the questionnaire was completed by the child’s father or mother at the time of data collection. There may be significant differences between fathers and mothers in terms of mindfulness, marital satisfaction, and coparenting, influenced by factors such as gender and social roles. In addition, studies have shown that children in some special families, such as single-parent families and reconstituted families, tend to be more prone to behavior problems [60]. Whether the results of this study can help special families requires further exploration in future studies.

Second, this study analyzed preschool children’s problem behaviors in aggregate, ignoring the uniqueness of each type of problem behavior. Future research could consider each dimension separately for a more in-depth analysis. 

Third, this study used parental reports to collect data, which may have been influenced by social approval effects. Future studies could adopt multiple subjects reporting children’s problem behaviors (e.g., teacher ratings) to make the data source more objective. 

Finally, developmental context theory emphasizes the temporal dimension in the ongoing interaction between the individual and the environment, and this interaction is age-specific depending on the individual’s stage of physical and mental development [61]. Therefore, future research could examine how parental mindfulness, marital satisfaction, and coparenting influence preschool child problem behaviors in the longitudinal dimension.

## 6. Conclusions

This study found that mindfulness negatively predicted preschool children’s problem behaviors. Both marital satisfaction and coparenting independently mediated the relationship between mindfulness and preschool child behavior problems. Marital satisfaction and coparenting play a chain mediating role between mindfulness and preschool child problem behaviors.

## Figures and Tables

**Table 1:** Demographic characteristics of the participants (N = 3448).

Characteristics (Code)	*n* (%)	Detection Rate of Children with Problems *n* (%)
Child’s gender		
Boy	1761 (51.1)	178 (10.1)
Girl	1687 (48.9)	141 (8.4)
Child’s grade		
Junior class of kindergarten	1193 (34.6)	114 (9.6)
Middle class of kindergarten	1010 (29.3)	93 (9.2)
Senior class of kindergarten	1245 (36.1)	112 (9.0)
Parent’s occupation		
A professional	402 (11.7)	34 (8.5)
Company administrator	334 (9.7)	29 (8.7)
Company employee	786 (22.8)	70 (8.9)
Service provider	329 (9.5)	30 (9.1)
Worker	201 (5.8)	28 (13.9)
Self-employed	496 (14.4)	41 (8.3)
Unemployed	251 (7.3)	25 (10.0)
Other	649 (18.8)	62 (9.6)
Only child		
Yes	1228 (35.6)	132 (10.7)
No	2220 (64.4)	187 (8.4)
Education level of the parent		
Junior high school and below	325 (9.4)	28 (8.6)
Senior high school	641 (18.6)	57 (8.9)
Junior college	847 (24.6)	83 (9.8)
Bachelor’s degree	1410 (40.9)	133 (9.4)
Master’s degree	210 (6.1)	17 (8.1)
Doctoral degree	15 (0.4)	1 (6.7)
Average monthly family income (CNY)		
Below 5000	596 (17.3)	69 (11.6)
5000~10,000	1298 (37.6)	124 (9.6)
10,000~15,000	766 (22.2)	66 (8.6)
15,000~20,000	384 (11.1)	26 (6.8)
More than 20,000	404 (11.7)	34 (8.4)

**Table 2:** Descriptive statistics and correlation analysis.

	M	SD	1	2	3	4	5	6	7	8	9
1 Parental mindfulness	72.98	12.03	1								
2 Marital satisfaction	21.09	7.98	0.37 ***	1							
3 Supportive coparenting	27.49	5.55	0.35 ***	0.71 ***	1						
4 Unsupportive coparenting	27.49	5.89	−0.30 ***	−0.64 ***	0.55 ***	1					
5 Coparenting	54.98	10.07	0.37 ***	0.76 ***	0.87 ***	0.89 ***	1				
6 Emotional problems	2.01	1.73	−0.27 ***	−0.25 ***	−0.18 ***	−0.30 ***	−0.27 ***	1			
7 Conduct problems	1.81	1.29	−0.27 ***	−0.28 ***	−0.27 ***	−0.29 ***	−0.31 ***	0.41 ***	1		
8 Hyperactivity	3.37	2.11	−0.30 ***	−0.29 ***	−0.30 ***	−0.26 ***	−0.32 ***	0.34 ***	0.44 ***	1	
9 Peer interaction	2.40	1.59	−0.20 ***	−0.23 ***	−0.20 ***	−0.26 ***	−0.26 ***	0.37 ***	0.28 ***	0.20 ***	1
10 Total score of behavior problems	9.59	4.79	−0.37 ***	−0.37 ***	−0.34 ***	−0.39 ***	−0.41 ***	0.74 ***	0.71 ***	0.75 ***	0.63 ***

Note: *** *p < 0*.001.

**Table 3:** Multiple regression analysis.

Regression Model		Regression Coefficient Significance				Overall Fitting Index		
Dependent variable	Independent variable	β	*t*	LLCI	ULCI	*R*	*R^2^*	*F*
Behavior problems	Gender	−0.41	−2.73 **	−0.70	−0.12	0.39	0.16	90.81 ***
	Grade	−0.20	−2.18 *	−0.38	−0.02			
	Only child	−1.08	−6.68 ***	−1.40	−0.76			
	Parents’ occupation	−0.03	−0.73	−0.09	0.04			
	Parents’ education level	−0.30	−3.68 ***	−0.46	−0.14			
	Average monthly family income	−0.19	−2.88 **	−0.32	−0.06			
	Parental mindfulness	−0.15	−23.32 ***	−0.16	−0.13			
Marital satisfaction	Gender	0.13	0.51	−0.36	0.62	0.39	0.15	88.88 ***
	Grade	0.28	1.82	−0.02	0.57			
	Only child	0.03	0.12	−0.50	0.56			
	Parents’ occupation	0.01	0.15	−0.10	0.12			
	Parents’ educational level	−0.60	−4.39 ***	−0.86	−0.33			
	Average monthly family income	−0.46	−4.17 ***	−0.68	−0.24			
	Parental mindfulness	0.25	23.60 ***	0.27	0.23			
Coparenting	Gender	0.01	0.03	−0.42	0.44	0.77	0.59	623.06 ***
	Grade	−0.18	−1.34	−0.44	0.08			
	Only child	−0.35	−1.50	−0.82	0.11			
	Parents’ occupation	0.07	1.40	−0.03	0.13			
	Parents’ education level	−0.19	−1.63	−0.43	0.04			
	Average monthly family income	0.25	2.54 *	0.06	0.44			
	Parental mindfulness	0.08	8.19 ***	0.06	0.10			
	Marital satisfaction	0.92	61.30 ***	0.95	0.89			
Behavior problems	Gender	−0.43	−3.01 **	−0.71	−0.15	0.49	0.24	122.04 ***
	Grade	−0.26	−3.06 **	−0.43	−0.09			
	Only child	−1.13	−7.36 ***	−1.43	−0.83			
	Parents’ occupation	−0.02	−0.55	−0.08	0.05			
	Parents’ education level	−0.23	−2.95 **	−0.38	−0.08			
	Average monthly family income	−0.09	−1.39	−0.21	0.04			
	Parental mindfulness	−0.10	−15.02 ***	−0.11	−0.08			
	Marital satisfaction	−0.05	−3.31 **	−0.02	−0.07			
	Coparenting	−0.12	−11.08 ***	−0.14	−0.10			

Note: *** *p* < 0.001, ** *p* < 0.01, * *p* < 0.05.

**Table 4:** Indirect effects of marital satisfaction and coparenting.

	Effect	Boot SE	Boot LLCI	Boot ULCI	Ratio of Total
Total indirect effect	−0.049	0.003	−0.056	−0.042	33.56%
Indirect effect 1 (*a*_1_*b_1_)	−0.011	0.004	−0.019	−0.004	7.53%
Indirect effect 2 (*a*_2_*b_2_)	−0.010	0.002	−0.013	−0.007	6.85%
Indirect effect 3 (*a*_1_*d*b_2_)	−0.028	0.003	−0.034	−0.022	19.18%

## Data Availability

The data presented in this study are available on request from the corresponding author.
